# PARTICIPANT EVALUATION OF 4MS EDUCATION DELIVERED BY AGE-FRIENDLY CARE, PA USING PROJECT ECHO: 2020–2023

**DOI:** 10.1093/geroni/igae098.2936

**Published:** 2024-12-31

**Authors:** Diane Berish, Jennifer Knecht-Fredo, Erica Husser, Donna Fick, Marie Boltz, Jacqueline Sabol, George Garrow, Angela Houge

**Affiliations:** The Pennsylvania State University, University Park, Pennsylvania, United States; The Pennsylvania State University, University Park, Pennsylvania, United States; The Pennsylvania State University, University Park, Pennsylvania, United States; The Pennsylvania State University, University Park, Pennsylvania, United States; The Pennsylvania State University, University Park, Pennsylvania, United States; The Pennsylvania State University, University Park, Pennsylvania, United States; The Primary Health Network, Sharon, Pennsylvania, United States; The Primary Health Network, Sharon, Pennsylvania, United States

## Abstract

Older adults, the largest segment of the US rural population, face significant disparities in health and healthcare compared to their non-rural peers. They have limited access to healthcare and social services for prevention, management, and treatment of chronic conditions. Age-Friendly Care, PA, a collaboration of Primary Health Network, the largest Federally Qualified Health Center in Pennsylvania, and Penn State Nese College of Nursing, aims to bring reliable, high-quality, age-friendly care to all older adults living in rural PA. Sponsored by HRSA through its Geriatric Workforce Enhancement Program, Age-Friendly Care, PA utilizes the Project ECHO (Extension for Community Health Outcomes), all-teach-all-learn, platform to engage isolated rural providers in incorporating the Institute for Healthcare Improvement’s 4Ms framework (What Matters, Mentation, Medication, Mobility) into their practice. Annual participant evaluation data from the last four years (2020 n=10, 2021 n=14, 2022 n=22, and 2023 n=6) shows respondents largely agreed or strongly agreed that attending the education session helped: Improve their ability to provide appropriate care (77%-100%), develop clinical knowledge (83%-100%), and 100% recommended attendance to colleagues. Participants were asked to rate their knowledge related to the 4Ms after each session. After the 2020 session, mobility screening, depression screening, and dementia training (66.6%) had the highest ratings. For 2021, depression screening (66.6%) was highest. In 2022, dementia screening and treatment (55%) were highest and for 2023, the top-rated knowledge area was treating mobility limitations (33.3%). Project ECHO has been shown to be an effective tool to reach rural healthcare providers and promote age-friendly education.

